# Infrared neural stimulation and inhibition using an implantable silicon photonic microdevice

**DOI:** 10.1038/s41378-020-0153-3

**Published:** 2020-06-01

**Authors:** Ágoston Csaba Horváth, Sándor Borbély, Örs Csanád Boros, Lili Komáromi, Pál Koppa, Péter Barthó, Zoltán Fekete

**Affiliations:** 1 Research Group for Implantable Microsystems, Faculty of Information Technology & Bionics, Pázmány Péter Catholic University, Budapest, Hungary; 2 Microsystems Laboratory, Institute for Technical Physics & Material Science, Centre for Energy Research, Budapest, Hungary; 3 Óbuda University Doctoral School on Materials Sciences and Technologies, Budapest, Hungary; 4 MTA TTK NAP Sleep Oscillations Research Group, Budapest, Hungary; 5 Department of Physiology and Neurobiology, Eötvös Loránd University, Budapest, Hungary; 6 Department of Atomic Physics, Budapest University of Technology & Economics, Budapest, Hungary

**Keywords:** Micro-optics, Applied optics

## Abstract

Brain is one of the most temperature sensitive organs. Besides the fundamental role of temperature in cellular metabolism, thermal response of neuronal populations is also significant during the evolution of various neurodegenerative diseases. For such critical environmental factor, thorough mapping of cellular response to variations in temperature is desired in the living brain. So far, limited efforts have been made to create complex devices that are able to modulate temperature, and concurrently record multiple features of the stimulated region. In our work, the in vivo application of a multimodal photonic neural probe is demonstrated. Optical, thermal, and electrophysiological functions are monolithically integrated in a single device. The system facilitates spatial and temporal control of temperature distribution at high precision in the deep brain tissue through an embedded infrared waveguide, while it provides recording of the artefact-free electrical response of individual cells at multiple locations along the probe shaft. Spatial distribution of the optically induced temperature changes is evaluated through in vitro measurements and a validated multi-physical model. The operation of the multimodal microdevice is demonstrated in the rat neocortex and in the hippocampus to increase or suppress firing rate of stimulated neurons in a reversible manner using continuous wave infrared light (*λ* = 1550 nm). Our approach is envisioned to be a promising candidate as an advanced experimental toolset to reveal thermally evoked responses in the deep neural tissue.

## Microsystems & nanoengineering neurobiology: heating and scanning neurons

Researchers in Hungary have developed a probe that can activate or inhibit neurons using infrared light while simultaneously measuring their cellular activity. A team led by Zoltán Fekete of Pázmány Péter Catholic University built and tested the device, which combines an optical waveguide to deliver the infrared light with wiring and measurement sites to record neural potential. The infrared beam heats a region of ~0.02 mm². The change in temperature can stimulate or inhibit neuronal firing, and the probe measures these changes in their behavior. The team tested the probe in the neocortex and hippocampus of rats and measured reversible changes in neural activity in both regions. The new photonic probe will enable researchers to investigate the effect of temperature fluctuations on neurophysiology and brain activity, including its role in neurodegenerative disease or brain injuries.

## Introduction

Temperature is undoubtedly an important factor in physiological processes^1^. The clinical relevance of its effect on several diseases has been recognized, however, we still have a limited knowledge on the spatial and temporal distribution and fluctuation of temperature, and its role in brain homeostasis. Besides the inherent thermal fluctuation in the brain, diagnostic and therapeutic tools may also introduce thermal changes in the tissue. Novel optical stimulation methods like optogenetics or semi-invasive imaging techniques relying on multi-photon excitation are hypothesized to cause significant heating of large volume of brain tissue during continuous illumination, increasing local temperatures by several °C^2,3^. Other groups have drawn our attention to the fact that related models often underestimate in vivo temperature rise during stimulation^4,5^. During electric stimulation of the spinal cord^6^ or stimulation with low-frequency magnetic field^7^, tissue heating is proposed to take into account when evoked responses are considered. Besides imaging artefacts of thermal origin, stimulation techniques relying on thermal intervention are also under intensive research, including infrared (IR) neural stimulation. Infrared neural stimulation (INS) uses pulsed IR light (between the wavelength of 1400–2100 nm) to generate highly controlled temperature transients in neurons (dT/dz or dT/dt), leading them to fire action potentials^8–10^. The main advantage of INS is that it can be applied to neural tissue without prior genetic incorporation of light sensitive opsins, thereby offering greater ease and flexibility of use^8^. In recent years, several applications of INS were published. It was shown that INS can alter GABAergic neurotransmission^11^, pace rabbit hearts^12^, activate specific regions in the brain (such as the visual cortex)^9^, and INS of auditory neurons was also presented^13^. Besides the excitatory effects of IR light, inhibitory effects have been discovered by Duke et al.^14^ who has demonstrated the suppression of neural activity in sciatic nerve fibres of a rat. Although the activation mechanism of INS relies on the spatiotemporal gradient of temperature, blocking of action potential propagation is supposed to be a function of an increase in baseline temperature^15^. Laser-induced inhibition is hypothesized to be caused by the non-uniform rate increases in temperature-dependent Hodgkin-Huxley gating mechanism, leading to reversible block of action potential generation^16^. The advantage of infrared neural inhibition (INI) compared with INS is that it requires significantly lower radiant exposure^17^, which is beneficial for the development of portable devices dedicated to investigations in behaving subjects. However, early results are promising, in vivo demonstration on intracortical or hippocampal targets has not been presented. The above studies focusing on both neurophysiology and neuroengineering confirm that there is still room for further investigation of temperature dependence as an experimental factor in vivo.

To gain more systematic information in this respect, multifunctional tools are desired, which provide parallel thermal stimulation and recording all integrated in microdevices of limited dimensions^18^. Optical stimulation microdevices or briefly optrodes, mostly dedicated to optogenetic manipulation, may provide alternative solutions to provoke thermal changes. An early hybrid system combining silicon microprobes with optical fibers has been proposed by Royer et al.^19^. Fabrication of integrated dielectric waveguides has been also utilized on top of implantable microelectrodes^20–23^. Besides passive optical components, the integration of light sources like laser diode die chips also offers new perspectives^24^. Probes holding addressable microLED arrays on the shaft have been also tested for in vivo use^25–27^. The operation of these devices are limited to the visible wavelength regime, however, near-IR applications are also gaining attention. Configurations of end-fire waveguide arrays^28^, Utah arrays^29^, and Michigan-type microprobes^30^ have been proposed to deliver IR light through embedded passive waveguides, but in vivo demonstration has not supported their safe and efficient operation. In this work, we demonstrate the in vivo use of an implantable multifunctional microsystem, which is able to deliver IR light and concurrently measure cellular activity in the deep neural tissue. The safe application of the proposed technique is established by systematic optical, thermal, and electrochemical characterization, which provides valuable input to introduce the device into customized experiments. To our knowledge, this is the first integrated microdevice used to perform deep tissue neuromodulation with IR light and simultaneously measures the evoked changes in firing characteristics of neurons.

## Results

### Design of integrated microdevice for thermal modulation of neural activity

**Fig. 1:**
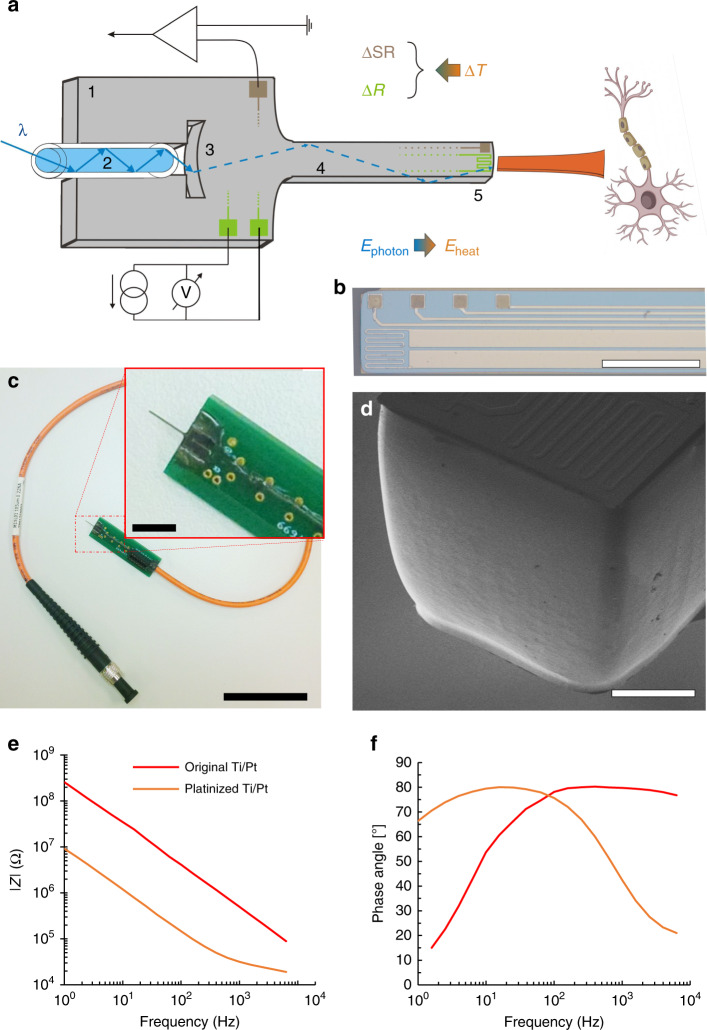
A silicon neural microprobe designed for infrared stimualtion/inhibition of neuronal firing and interrogation of extrcellular potentials and temperature. **a** Components of the implantable microsystem. 1: Si substrate, 2: multimode optical fiber, 3: cylindrical coupling lens, 4: shaft with multiple functionality, 5: probe tip. Figure is not to scale. **b** Optical microscopy image of the optrode tip. (Scale bar shows 300 µm) **c** Photo of an assembled optrode device. Scale bar of the picture with small and larger magnification are 3 cm and 5 mm, respectively. **d** Scanning electron micrograph representing the surface quality of the optrode tip. (Scale bar shows 100 µm) **e**, **f** Amplitude and phase diagrams of the impedance of the electrophysiological recording sites, respectively

Our approach to investigate thermal modulation of neural activity relies on a multifunctional probe design, which provides stimulation and measurement functions simultaneously. This advanced microsystem is manufactured from single-crystalline silicon, and comprises integrated photonic and electrical components. The configuration is shown in Fig. 1a. The probe holding an embedded waveguide is connected to an optical fiber delivering IR light (*λ* = 1550 nm) from a pig-tailed laser diode (max. power: 70 mW). The 5 mm long, 170 µm wide, and 190 µm thick shaft of a silicon probe acts as an optical waveguide and carries conductive and insulating thin film layers that forms wiring and sites for recording neuronal action potentials and changes in tissue temperature. The transmission of IR light is facilitated by a wet chemical nanomachining process detailed in our prior work^31^. The resulting surface roughness of the silicon shaft makes low-loss delivery of IR light into the tissue (see Fig. 1d). The coupling of IR light from an external light source (e.g., laser diodes) is guided through integrated microstructures. The IR radiation exits the shaft at the tip, and is eventually absorbed in the tissue. The average overall efficiency of the microoptical system delivering the IR light at chip-scale is measured as 32.04 ± 4.10%, whereas the max. efficiency in packaged form is 41.5 ± 3.29%^30^. The average beam spot size at the probe tip is 0.024 ± 0.006 mm^2^. The absorbed energy is converted to heat, which can be measured using integrated platinum temperature sensor during device operation to maintain stable temperature in the vicinity of the modulated population of neurons. The evoked neuronal response like action potentials can be monitored through embedded platinum recording sites located close to the end facet of the waveguide (see Fig. 1b). The chip device is mounted on a custom printed circuit board, which provides stable connections to electrical and optical sockets (see Fig. 1c). More details on micro- and nanofabrication and results on the optical characteristics (like beam characteristics, optical profile) of the system can be found in our prior work^30^. The overview of the microfabrication steps is shown in Fig. S1. The validated optical model of the microsystem has been also published by our group^32^. In the current work, thermal characterization and in vivo validation of the device is presented. The optical efficiency of the device presented in this paper is 24.2 ± 6.9% (*n* = 5). The sensitivity of the integrated temperature sensor is TCR = 2636 ± 75 ppm/K (*n* = 5). The electrochemical characteristics of the recording sites are shown in Fig. 1e, f. Beyond the preparation steps of the device already presented in our prior work^30^, the impedance of the recording sites is further reduced by depositing porous platinum on the sputtered platinum surfaces. This extra electrochemical process reduces the initial impedance value at 1 kHz decreased from 678 ± 198 kΩ to 46 ± 9 kΩ (*n* = 4). Deposition method is described in the Electrochemical characterization section.

### Distribution of temperature in continuous mode control

**Fig. 2:**
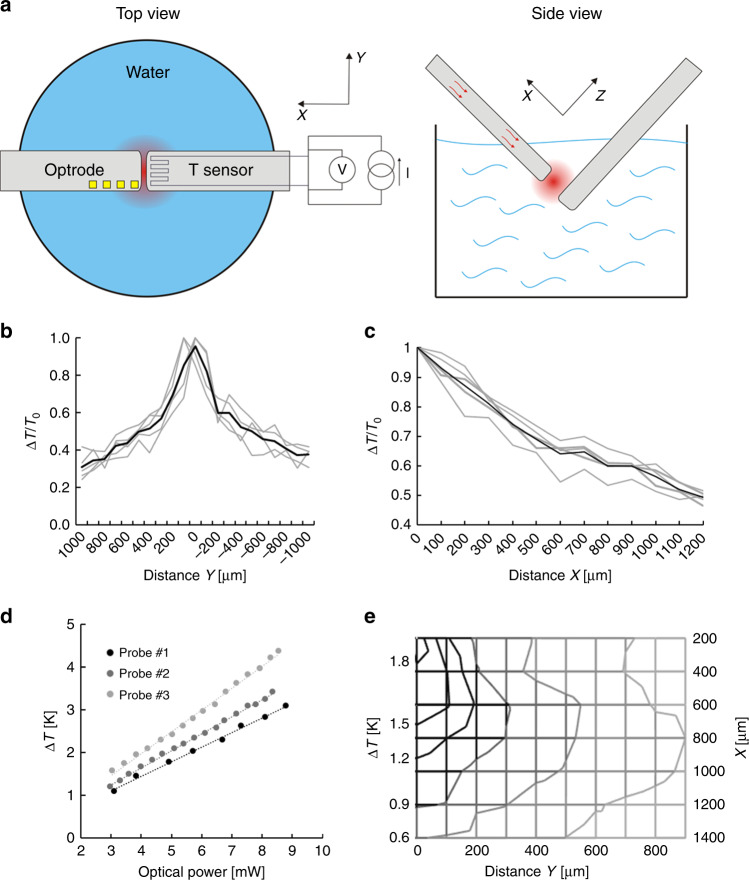
Thermal characteristics of the integrated microdevice. **a** Schematics of the in vitro experimental setup for the characterization of spatial temperature distribution. **b** Normalized distribution of the temperature increase along the *y* axis (*x* = 200 µm). **c** Relative rise in temperature as a function of distance between the tips of the immersed optrode and the external temperature sensor. **d** Calibration curves: optical power vs. rise in temperature. **e** 2D representation of the distribution of temperature rise along two perpendicular axes *x* and *y*. The location of the probe tip is considered the origin of the coordinate system

For the safe use of the proposed technique, it is essential to determine the spatial distribution of temperature around the probe tip, precisely. An in vitro experimental setup is prepared (schematic in Fig. 2a), where the optrode under investigation is immersed in water, and temperature in the surrounding medium is monitored through a calibrated external platinum temperature sensor. The setup allowed the positioning of the external temperature sensor with respect to the optrode along multiple axis. These data provide valuable information on the decay of heat energy accumulated during the absorption of IR radiation in the wet medium (Fig. 2b, c). The full width at half maximum of the distribution of local radiant heating is 1020 ± 184 µm along the *y* axis (*x* = 200 µm) perpendicular to the shaft. More experimental details can be found in the Methods section. Reconstruction of a 2D distribution of a particular probe is shown in Fig. 2e.

Before in vivo experiments, a calibration procedure was performed to determine diode specific operational characteristics (see optical power vs operating current in Fig. S2) and rise in temperature as a function of optical power (see Fig. 2d). These curves were considered during the design of each animal experiment. To check the reliability of the integrated temperature sensor, the relationship between integrated and external temperature sensor (at location *x*(*y* = 0) = 200 µm) was measured (Fig. S3a) and calibrated through a fiber-optic temperature sensor (Fig. S3b) to ensure the proper interpretation of the data on temperature. It was found that the external measurement gives a higher estimation of temperature than the integrated sensor by 24 ± 6%, so we rescaled our measured data in further in vivo use. As the probe can be mounted with optical fibers of various core diameter, the effect of core diameter on temperature distribution was also checked along the *y* axis (Fig. S4). There is a slight change in the shape of the distribution, which may be attributed to the change in the beam shape. More details on the effect of fiber core diameter and beam shape on the optical output can be found in our prior work^33^.

### Thermal modulation of spiking activity

**Fig. 3:**
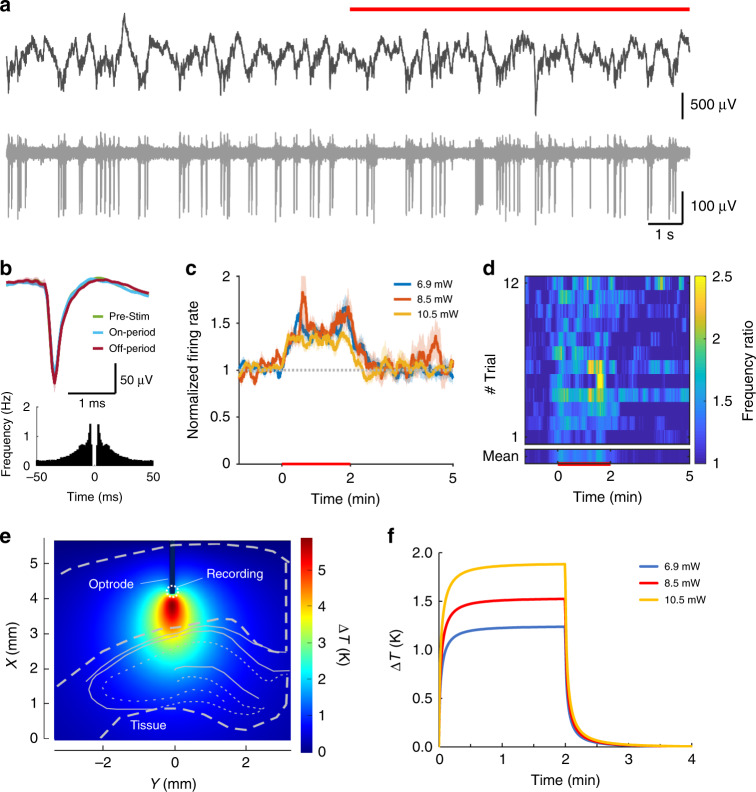
Infrared excitation of cortical neurons. **a** Local field potential (top) and unit activity (bottom) at 1300 μm cortical depth recorded from the multimodal probe before and during (red line) heating. **b** Waveform and auto-correlogram of a single unit clustered from the recording site on **a**. The spike waveform does not change between the pre-stimulus, stimulus-on, and stimulus-off periods. **c** Changes in firing rate of cortical multiunit for various excitation powers. **d** Average spike rate difference across trials (heat map). **e** Simulated temperature distribution during stimulus onset around the excited region at 10.5 mW and **f** simulated temporal change of temperature in the vicinity of the integrated temperature sensor of the optrode in the case of all applied optical power in this experiment

**Fig. 4:**
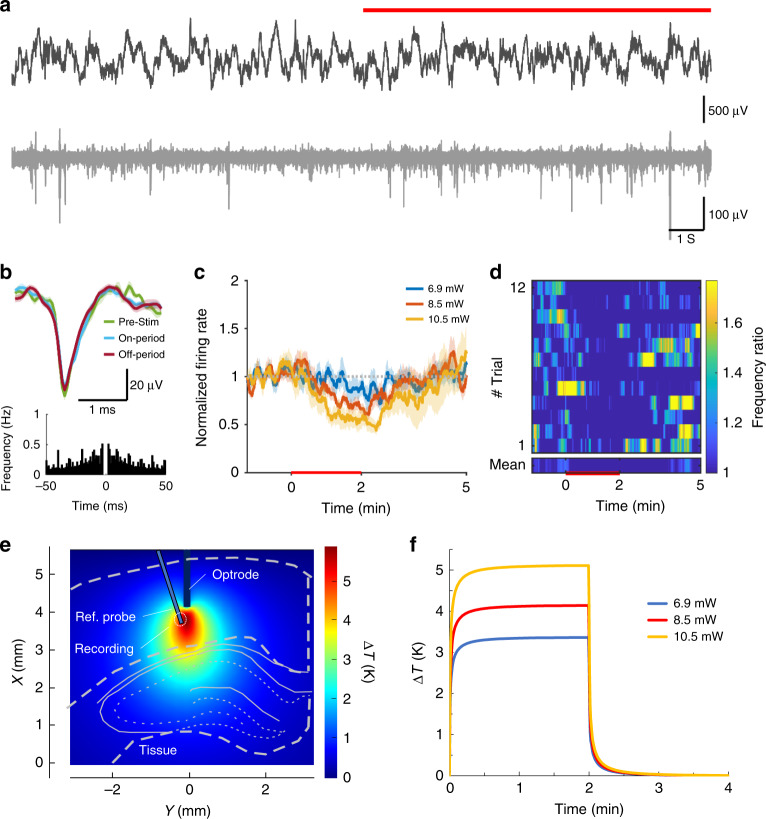
Infrared inhibition of cortical neurons. **a** Local field potential (top) and unit activity (bottom) recorded from the 16-channel silicon probe at 1600 μm cortical depth before and during (red line) heating. **b** Waveform and auto-correlogram of a single unit clustered from the recording site on **a**. The spike waveform does not change between the pre-stimulus, stimulus-on, and stimulus-off periods. **c** Changes in firing rate of cortical multiunit for various excitation powers. **d** Average spike rate difference across trials (heat map). **e** Simulated temperature distribution during stimulus onset around the excited region at 10.5 mW and **f** simulated temporal change of temperature in the vicinity of the integrated temperature sensor of the optrode in the case of all applied optical power in this experiment

**Fig. 5:**
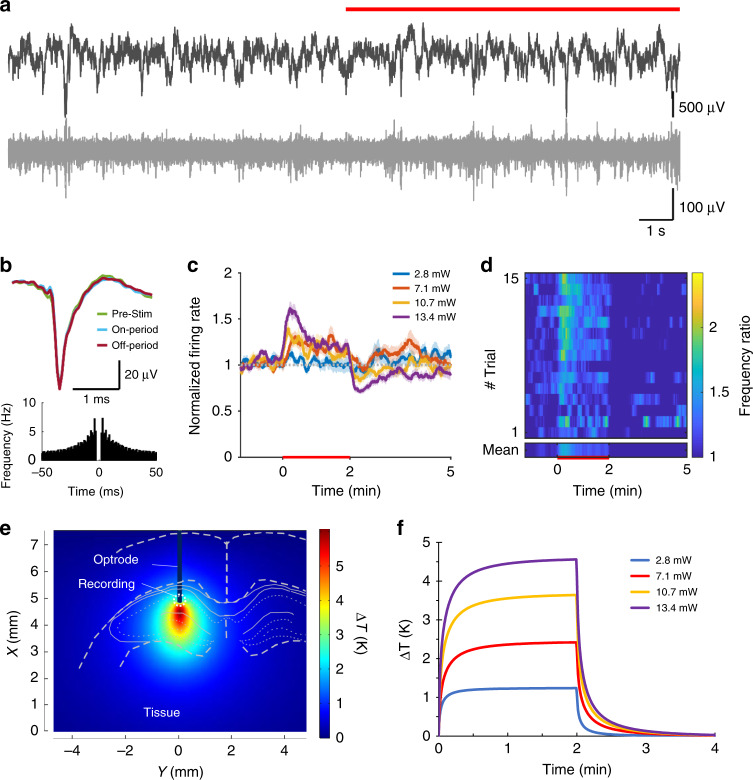
Infrared excitation of hippocampal neurons. **a** Local field potential (top) and unit activity (bottom) recorded from the multimodal probe before and during (red line) heating. **b** Waveform and auto-correlogram of a single unit clustered from the recording site on **a**. The spike waveform does not change between the pre-stimulus, stimulus-on, and stimulus-off periods. **c** Changes in firing rate of hippocampal multiunit for various excitation powers. **d** Average spike rate difference across trials (heat map). **e** Simulated temperature distribution during stimulus onset around the excited region at 13.4 mW optical power and **f** simulated temporal change of temperature in the vicinity of the integrated temperature sensor of the optrode in the case of all applied optical power in this experiment

Experiments in rat subjects were performed to test electrode functionalities and to interrogate evoked responses to IR neuromodulation in the cortex and in the hippocampus. Fig. S6 shows the experimental arrangement of surgery using two implanted devices: our multimodal device and a laminar silicon probe as control for the validation of recorded data. In our stimulation protocols comprising 2 mins ON and 4 mins OFF periods, the change of spiking activity of neurons in the vicinity of the irradiated regions was evaluated using various optical powers at a wavelength of 1550 nm. At least 10 trials per optical power in a randomized fashion were recorded to determine the characteristic changes in neuronal firing pattern. In the raw data, no visible artefact owing to stimulus onset was detected (Figs. 3a, 4a and 5a). To validate our coupled optical–thermal model, concurrent measurement of tissue temperature was also carried out with the integrated temperature sensor at various optical powers (see Fig. S12). Action potential waveforms were not affected by the stimulation, even after several hours (Figs. 3b, 4b and 5b), indicating the lack of neural damage. The spatial distribution and temporal dynamics of the rise in temperature induced by the IR irradiation at the location of single unit recording were estimated by the multi-physical model. Related data were visualized for each experimental arrangement (see Methods section). Figures 3–5 summarize our findings for cortical excitation, cortical inhibition, and hippocampal excitation, respectively.

Cortical multiunit activity at 1300 μm showed a significant increase at all optical power values (*p* < 0.01 for 6.9, 8.5, 10.5 mW, Fig. 3c, d). Multiunit activity at 1600 μm, however, was suppressed throughout the stimulus at high optical power (*p* < 0.01 for 6.9, 8.5, 10.5 mW, Fig. 4c, d). A similar protocol in the hippocampal CA1 pyramidal layer caused an increase in multiunit activity at high power stimuli (*p* < 0.01 for 2.8, 7.1, 10.7, 13.4 mW, Fig. 5c, d).

Figure S9 contains representative data on the change in body (rectal) temperature during the stimulus sequences, which also supports our claim that no global changes are in correlation with the local rise in brain temperature during the course of experiments.

## Discussion

It is well established that temperature has influence on several neurophysiological properties including resting potential, action potential, and synaptic transmission^34^. In view of the efficiency of cellular metabolism, the overall thermal impact on cell population is apparent. Approximately, 40% of the energy produced during oxidative metabolism is used to convert glucose to ATP in the brain, whereas 60% is dissipated as heat^35^. This metabolic activity is still high in resting states. Metabolism through the blood–brain barrier is also intensified through larger permeability in response to temperature increase^36^. The advances in brain diagnostics helped us to recognize that pathological hyperthermia is a modulatory factor in brain disorders^37^. As a result, hypothermia has been proposed as a therapeutic tool to limit neuronal injury in neurodegenerative diseases. The efficiency of hypothermia has been proven in cases of ischemia^38^, epilepsy^39^, and traumatic brain injury^40^. Underlying processes are intensively researched at various level of organization in the brain. At the synapse level, Brownian motion governs the diffusion of molecules (like neurotransmitters and calcium ions) and has potential effect on the motion of proteins within the membrane or the cytoplasm^41^. Horvath et al.^42^ revealed first that synaptic transmission is also sensitive to heat in the homeostatic center of the brain. Their results received gradually increasing attention as TRP channels have been discovered, and have given evidence to the modulatory effect of temperature in cellular communication^43^. Some of these thermally activated receptors are active at normal physiological temperature as well^44^. At the cellular level, response to elevated temperature may change the excitability of cells, which is substantiated by the occurrence of spontaneous action potentials and increased spontaneous synaptic activity^45^. This cellular response can be definitely characterized using implantables, dedicated to (1) induce thermal changes in a spatially confined manner and (2) record brain signals in a single cell level. In our work, characterization and in vivo validation of such an implantable photonic microdevice was demonstrated. Description of spatial distribution of temperature induced by IR excitation will definitely provide guidelines for future in vivo use. Optical–thermal models validated in real experiments will also definitely help the evaluation of more complex experiments focusing on the evoked response of several brain regions to locally controlled thermal changes. Our results confirmed that, changes in the spike rate of both cortical and hippocampal neurons using our integrated approach are reversible, and there is no influence on either waveforms of individual neurons, oscillatory activity, or body temperature. This means that the effective cross-section of our stimulation procedure is small enough to avoid direct global changes in physiological mechanisms.

Our primary aim was to test concurrent IR stimulation and electrical recording in the deep neural tissue. Our study is the first in vivo test with such photonic device. Similar work in the literature has demonstrated Utah configuration for addressing intracortical IR stimulation, however, the authors have not proved in vivo performance^29^. Regarding the quality of neural signals recorded with our probe and the reference laminar probe, we found that no photoartefacts induced by IR stimulation can be detected during signal acquisition. This is especially important in view of the well-known Becquerel effect deteriorating electrical signals during optogenetic stimulation with visible light^46^. Using the integrated temperature sensor located on the probe shaft, we are also able to monitor the change in temperature, which provides a valuable feedback helping us to ensure safe operation of the device. In contrast to optogenetic methods, IR neural stimulation uses water as the chromophore, no viral sensitization is needed. In the extracellular medium, IR light is converted into heat, and owing to conductive heat propagation has a much larger effective cross-section (or stimulated volume of tissue in other words). For this reason, the spatial configuration of the end facet of the waveguide is not as critical as for devices designed to deliver visible light, where stimulation sites should be located in the close vicinity of the recording sites.

Our secondary aim was to reveal potentials of the technique in vivo, which may initiate new approaches to investigate debated hypotheses with a novel experimental toolset. For this reason, the recording depth was varied to cover regions from the superficial layer of the cortex down to the CA1 region of the hippocampus. In layer V of the cortex, a permanent increase in firing rate was observed without apparent sensitivity to the change in temperature above a threshold in the optical power range of (5–15 mW). In parallel with this example of neuronal excitation, inhibitory effect was also detected in layer VI, which was proportional to the change in temperature. The rise and recovery time in this case (~3 minutes) was substantially longer than that of the excitatory response (<1 minute). Approximately 50% change in the spike rate was achieved in both cases, when the highest excitation power was applied. In the hippocampus, variations in the frequency of multiunit activity was found to be similar to the data on cells recorded in the supragranular layers of the cortex. In this region, sensitivity to temperature gradient was more remarkable than that measured in the cortex.

Some recent studies in the literature has shown similar observations, however, these experiments were limited to in vitro subjects^44,46–50^. For example, in our experiments, the activity of CA1 neurons was recorded during in vivo stimulation. There is a growing literature debating the expression, presence, and function of thermosensitive receptors and ion channels in the hippocampus^45,51^, however, the in-depth investigation of the underlying phenomena and sensitivity to temperature was out of the scope of our work. Nevertheless, the very recent results of Xia et al.^50^ suggests that safety limits are far beyond the range we used, therefore our toolset is definitely able to address questions on cell excitability modulated with tissue temperature.

Besides the above works on the response of brain cells to hyperthermia, studies on INS and INI may also benefit from the use of our photonic microdevice. In particular, our results indicate that low-energy (in the range of a few mW) irradiation of the intracortical and hippocampal neurons is able to either boost or suppress the firing activity of neurons without creating high spatial or temporal gradient of temperature increase. Nevertheless, the degree of inhibition (decrease in firing rate) in our case of infragranular cells are in the same range as demonstrated in vitro by Xia et al.^50^ at 1550 nm with continuous wave laser light and in vivo by Cayce et al.^52^ at 1875 nm with pulsed IR irradiation.

## Methods

### Fabrication and design

Fabrication of the silicon optrode device relies on standard MEMS fabrication technology (Fig. S1). The fabrication process is described in details in ref. ^30^. The ready-to-use device is shown in Fig. 1c. The length of the optrode shaft is 5 mm, and width and thickness are 170 µm and 190 µm, respectively. The wires and recording sites located on the silicon carrier are composed of Ti/Pt multilayer (total thickness: 270 nm) embedded in a silicon-dioxide/silicon nitride dielectric stack (total thickness: 600 nm). The sidewalls of the fabricated optrodes are optimized through wet chemical etching to be optically smooth. This is necessary as deep reactive ion etching, used to release probe structures from the silicon wafer, results in periodic grooves of high roughness throughout the sidewall surfaces (see our prior work in ref. ^31^ for detailed analysis). The optically smooth sidewalls (see Fig. 1d) facilitate total internal reflection of IR light inside the probe shaft. As a result, IR light can be coupled into the 5 mm long Si shaft and can be delivered into the neural tissue. The illuminated region is the probe shaft, in this case 170 µm × 190 µm. IR light (wavelength of 1310 or 1550 nm) is guided through a multimode glass optical fiber butt-coupled to the Si chip (see Fig. 1a). In the current design, a laminar array (intersite distances of two types were 100 µm and 300 µm) of four recording sites and a platinum filament as calibrated temperature sensor are located on the probe tip. The sites and the filament are connected to a custom designed PCB to record neuronal activity and temperature.

### Electrochemical characterization

The impedance of each platinum recording sites was characterized using electrochemical impedance spectroscopy (EIS). A Gamry Reference 600 Potentiostat (Gamry Instruments, Warminster, PA, US) was used in a three-electrode compartment filled with 0.01 m phosphate-buffered saline solution (P4417, tablet diluted in 200 mL distilled water, Merck KGaA, Germany). A leakless miniature Ag/AgCl electrode (ET072-1, eDAQ Pty Ltd., Australia), a Pt wire and the recording site of the optrode were applied as reference, counter and working electrodes, respectively. 25 mV rms voltage was utilized for sweeping between a frequency range from 10 Hz to 10 kHz. All of the electrochemical measurements were conducted in a Faraday-cage at room temperature in a cleanroom environment.

To further reduce the impedance of the platinum recording sites, we improved the active surface area by electroplating porous platinum (also called as black platinum). The same potentiostat was applied as for EIS, in galvanostatic mode. For the deposition of the porous layer, lead free 1 wt.% chloroplatinic acid solution (diluted from 8 wt.% chloroplatinic acid solution in H_2_O, Merck KGaA, Germany) was used along with PVP (Polyvinylpyrrolidone, Merck KGaA, Germany) to improve the wettability of the sputtered platinum surfaces. The deposition processes were carried out in a threecompartment electrochemical cell. A leakless miniature Ag/AgCl (3.4 mol/L KCl) electrode (ET072-1, eDAQ Pty Ltd., Australia) and a platinum sheet were used as reference and counter electrode, respectively. Recording sites have been electroplated by maintaining current density of 10 mA/cm^2^ for 60 s. This individual deposition approach reduced the variability of site impedances as low as possible. Gamry Echem Analyst software (Gamry Instruments, Warminster, PA, US) was used to evaluate and represent the Bode plot of the measurement. Fig. 1e, f show a comparison of the impedance magnitudes and phase angles of the recording sites before and after electroplating.

### Characterization of absolute output power

To determine the absolute optical power exiting the probe tip, we performed optical measurements with a Germanium IR sensor (OP-2 IR, Coherent Inc, CA, USA) connected to a laser power meter (FieldMaxII-TOP, Coherent Inc, CA, USA). During the course of the in vitro experiments, two light sources have been used. A flat-window IR laser diode (RLT1300-40G, Roithner GmbH, Austria) with a nominal optical power of 40 mW and a wavelength of 1.31 µm was applied to conduct initial testing of the setup for thermal measurements. In this case, the beam of the laser diode was focused onto the core of the multimode optical fiber through a custom designed optical bench described in our prior work^30^. Later on, the characteristic spatial distribution and calibration curves were acquired by using a pig-tailed laser diode (LPSC-1550-FG105LCA-SMA, Thorlabs, Inc., USA), which provided a nominal power of 50 mW at a wavelength of 1.55 µm.

During beam power measurement, the optrode device was fixed on a 1D translation stage facing the laser power meter without any imaging optics (Fig. S8/B). The tip of the optrode was positioned to the axis of IR detector and 100 µm away from its active surface (cf. Fig. S8/A). During optical heating measurements and in vivo experiments, the laser diode was driven with different DC currents using a Keithley 2635 A source measure unit (Keithley Instruments Inc, OH, USA). We measured and recorded the output optical power of each optrode at all driving currents in the operation range of the diodes to determine calibration curves. Representative characteristic curves can be seen on Fig. S2. These measurements of absolute power were systematically repeated during the course of in vivo experiments to avoid misinterpretation of the data owing to possible degradation in the light source or in integrated components of the probe. In contrast to several publications on optical stimulation, we used absolute optical power throughout the text instead of power density or energy density, however, both can be derived easily as the area of the stimulation site was fixed (0.024 ± 0.006 mm^2^).

### Characterization of output heat distribution

Testing of optical heating was performed in saline in 1.7 ml compartment at room temperature. The schematic of the measurement setup is shown in Fig. 2a. The shaft of two Si probes were immersed in the liquid medium. One was the heating source (optrode) and another one was used to measure the temperature change in different positions from the end facet of the optrode (reference point of the coordinate system). IR light was coupled to the optrodes from the same light source used for absolute optical power measurement. This radiation was emitted from the probe tip and was absorbed in the water medium, therefore elevated the temperature of that. This change in temperature at the illumination spot was recorded by an external temperature sensor (*R*_0_ = 358.56 Ohm, TCR = 2650 ppm/K, size: 100 µm × 100 µm) located on the tip of the other Si probe. The spatial change of temperature was recorded at multiple locations along the axis of the shaft (*x*) and also in perpendicular direction (*y*) with a step-size of 100 microns set by a micropositioner. The optical power is derived from the absolute optical output power measurements. The excitation waveform is presented in Fig. S9.

### Thermal modeling

In our previous work, a coupled optical–thermal model of the optrode device was developed and validated by surface topographic and optical measurements^32^. In the present study, the validation of thermal results of the model was carried out by temperature measurements discussed in section 2.2. The thermal model developed in COMSOL Multiphysics uses the optical intensity distribution at the probe tip provided as an output of a ray tracing model implemented in Zemax^32^. As a result, the absorbing liquid medium at the tip is the heat source in our thermal model, and the temperature distribution is calculated using the heat transfer equation. The material properties as heat capacity, density, thermal conductivity were set for silicon and water from the material library of COMSOL. The outputs of the simulation are the spatial and temporal evolution of the temperature distribution. In the simulated configuration, the shaft is immersed in a depth of 3 mm into the liquid medium (water) kept in the polyethylene cylinder, which is also taken into account during heat conduction. The radius and the height of the cylinder is 6.7 mm and 12 mm, respectively, the thickness of the wall is 1 mm. The excitation peak value, periodic time and pulse length were 7.88 mW, 120 s, and 60 s, respectively. The spatial distribution along the *X* and *Y* direction from the base position (see Fig. 2a) are shown on Fig. S10A and B in equilibrium state. The temporal changes are represented in Fig. S11. The temperature values provided by the thermal model were also validated through in vivo measurement (see a representative curve in Fig. S12).

### Surgery

Experiments were made in accordance with the Hungarian Act of Animal Care and Experimentation (1998, XXVIII) and with the directive 2010/63/EU of the European Parliament and of the Council of 22 September 2010 on the protection of animals used for scientific purposes. Experimental protocol was approved by the regional ethical committee (license number PEI/001/2290-11/2015 for our in vivo experiments). Efforts were made to minimize the number of animals used. Rats were kept under a 12:12 h light–dark cycle (lights-on at 7:00 a.m.) in a temperature-controlled room at 22 ± 2 °C. Standard food-pellets and tap water were available ad libitum.

Our acute experiments were carried out on three male wistar rats (Toxicoop, Budapest, Hungary) weighing between 230 and 440 g at the time of the surgery. The animals were anesthetized with urethane (1 g/kg; i.p.), then placed in a stereotaxic instrument (RWD Life Science; Shenzhen, China). A single large craniotomy was made over the somatosensory cortical region. After durotomy, the electrodes were inserted and we waited at least for 30 minutes before recording.

### Electrophysiology recordings

To record local field potential (LFP) activity, NeuroNexus (Ann Arbor, USA) silicon probes were inserted into the brain. Linear 16-channel silicon probes were inserted to the lateral part of left somatosensory cortex (Br. AP −3.3, L + 2.7, in 18° at 1800 or 2800 μm depth in the case of cortical or hippocampal recordings, respectively., while our custom designed optrode, featuring four electrophysiological recording sites, a platinum temperature sensor and a built‑in light cable, was inserted to the medial part of somatosensory cortex (Br. AP −3.3, L + 2, in 0° at 1600 or 2600 μm depth in the case of cortical or hippocampal recordings, respectively). An additional screw electrode implanted over the cerebellum served as a reference. Coordinates are based on the stereotaxic atlas of Paxinos and Watson (1986).

Recordings were made with Intan RHD2132 16-channel amplifiers, connected to an RHD-2000 Evaluation Board. Rectal temperature was measured by a TH-5 Thermalert Monitoring Thermometer (Physitemp; Clifton, USA) and recorded using the analog inputs of the Intan RHD-2000 system. All signals were sampled at 20 kHz. Long-term heating sessions were made by laserdiode system (LPSC-1550-FG105LCA-SMA, Thorlabs, Inc., USA) connected to our custom optrode and controlled by Keithley 2635 A device (Keithley Instruments Inc, OH, USA). Heating was triggered by a square pulse generated by an NI-USB 6211 (National Instruments, TX, USA) data acquisition system, connected to x86-based PC, which was also used for LFP recording simultaneously.

### Evaluation of electrophysiology

Raw LFP channels were band pass filtered between 0.4 and 7 kHz, and multi-units were detected with an absolute threshold. The unit activity was combined from multiple neighboring channels, downsampled to 1 kHz and smoothed with a 10 ms moving average filter. These data were used for calculation of peri-stimulus time histogram of heating events. Single unit detection was made by a simple thresholding method, followed by a manual clustering. As we always detected only one cluster, we used this method only for removing artefacts and unexpected sudden noise.

### Optical stimulation protocol

Optical output power of each probe was measured before and after the in vivo experiment to check beam stability and ensure validity of the evoked responses. For each animal, the stimulation cycle was composed of 2 min long ON (diode switched on), and 4 min long OFF (diode switched off) periods to allow sufficient time for the temperature of the stimulated region to return to baseline temperature. To check the reproducibility of the stimulation patterns in the electrophysiological traces, 10–15 trials were performed in a random fashion for each applied power (temperature). Driving current of the IR laser diode was supplied by a Keithley 2635 A source measure unit (Keithley Instruments Inc, OH, USA). Each onset of the laser diode was synchronized with trigger signals generated by an NI 6211 Data Acquisition Board (National Instruments, Austin, TX, USA).

## Supplementary information

Editorial Summary

## Data Availability

The data sets generated during and/or analyzed during the current study are available from the corresponding author on reasonable request.
