# Supplementary material for: Infrared neural stimulation and inhibition using an implantable silicon photonic microdevice
Source: Microsyst Nanoeng. 2020 Jun 1;6:44. doi: 10.1038/s41378-020-0153-3 (PMC8433474; doi:10.1038/s41378-020-0153-3)
Supplement: Supplementary file 1 — Editorial Summary [file 41378_2020_153_MOESM1_ESM.docx]

# Supplementary

Figure S1


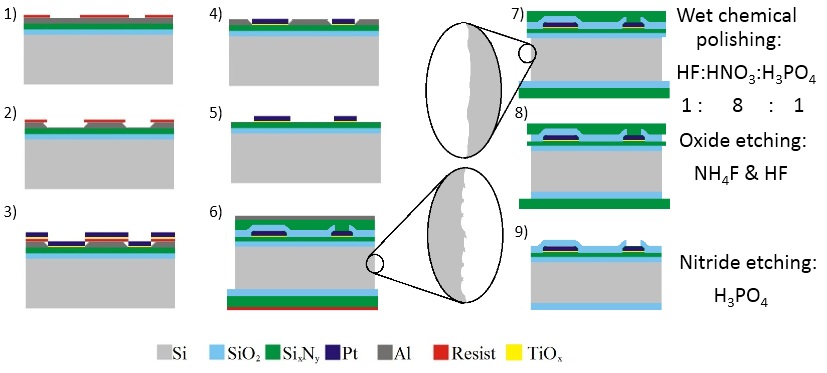


*Figure S1. Schematic representation of technology process flow of optrode fabrication. Three post-processing steps were developed in (Kiss, 2016) to transform the shaft of a standard silicon neural probe into low-loss infrared waveguide. The whole manufacturing scheme is detailed in our prior work^30^*

The fabrication of the multimodal silicon probe relies on silicon MEMS (Micro-electro-mechanical systems) processes. The initial substrate is a 200 µm thick (100) single-crystalline silicon wafer. Double-side polished wafers are used to maximize the efficiency of the integrated Si waveguide by maintaining the atomic smoothness of the wafer surface. Wet oxidation of silicon wafers is performed at 1100 °C. 50 nm thick thermal SiO_2_ layer is grown on the substrate surface. To further isolate the recording sites from the bulk Si, a 300 nm thick low-stress silicon nitride (SiN_x_) film was deposited in an LPCVD chamber (Tempress Systems, Inc., Netherlands) at 830 °C and 200 mTorr. The gas flow rate of H_2_SiCl_2_ and NH_3_ was 160 sccm and 20 sccm, respectively. The SiN_x_ and SiO2 are removed from the backside of the wafer by wet etching in phosphoric acid and diluted HF, while front side layers are protected by low temperature oxide and SPR 4.0 photoresist (Figure S1, step 1). Wet etching was used instead of dry etching to maintain the smoothness of the backside surface. A sacrificial Al layer was used to define the pattern of the TiO_x_/Pt recording sites, temperature monitoring filament and wires via a standard lift-off process (step 2). First, a 300 nm thick sacrificial Al layer was deposited by electron beam evaporation. This was followed by the first photolithography step using Microposit 1818 photoresist (Rohm and Haas Company, USA), and etching steps defining the inverse pattern of the conductive layers. The conductive layers consisted of a 15 nm thick adhesion layer of TiO_x_ formed by reactive sputtering of Ti in an Ar/O_2_ atmosphere (Ar/O_2_ ratio was 80:20). 270 nm thick Pt was sputtered on top of TiO_x_. The deposition of the two layer was performed in a single vacuum cycle using a DC Magnetron sputtering equipment (Leybold GmbH, Germany) (step 3). To complete the lift-off process step, photoresist and Al were removed in acetone and in nitric acid, respectively (4-5). In the next fabrication step, the top passivation layer of 1000 nm thick SiO_2_ layer was deposited using LPCVD at 430 °C in a gas mixture of SiH_4_ & O_2_. Contact and bonding sites were exposed by additional photolithography (Microposit 1818 photoresist) and dry etching step through a 100 nm thick aluminum hard mask (step 7). Dry etching of the SiO_2_/SiN_x_/SiO_2_ dielectric stack was performed in an Oxford Plasmalab 100 DRIE chamber using fluorine chemistry (step 8). 200 nm SiN_x_ as a masking layer for wet chemical polishing was deposited again in the LPCVD system using the above parameters (step 6). The probes were then micromachined by dry etching using Bosch recipe in an Oxford Plasmalab System 100 DRIE chamber (Oxford Instruments Plc, UK). Masking layer was e-beam evaporated aluminum on the front side, while bottom SiO_2_/SiN_x_ stack acted as etch stop layer. A protective 4 micron thick photoresist layer was also utilized on the backside of the wafer (step 7). Chamber pressure was 30 mTorr. ICP power was set to 750 W. C_4_F_8_ and SF_6_ flow rate were 100 sccm and 150 sccm, respectively. The duration of etch and passivation cycles were 4 and 9 seconds, respectively. Finally, masking layers were removed in acetone (step 8). Fluorocarbon polymer inherently deposited on the trench sidewall during Bosch-process was removed in a high-temperature oven at 600°C in O_2_. The planarization of the probe sidewall is achieved by immersing the wafer in a polishing mixture of HF : HNO_3_ : H_3_PO_4_ in a ratio of 1 : 8 : 1 at 20 °C for 2.5 minutes (etch rate: 9-10 µm/min). The low-temperature oxide membrane at the bottom of the trenches is dissolved in buffered oxide etchant, then top and bottom SiNx protective layer is also removed completely in phosphorous acid (step 9).

Figure S2


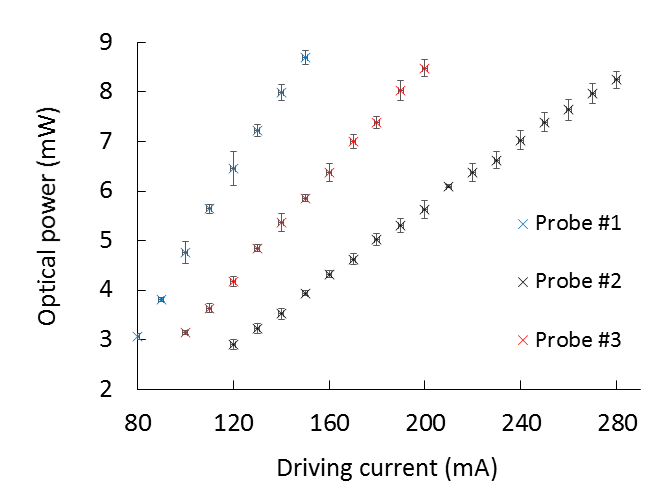


*Figure S2. Characteristic change in optical power vs. driving current of laser diodes (average + SD for three different optrodes). Measurements were performed at the probe tip using an absolute power meter.*

Figure S3


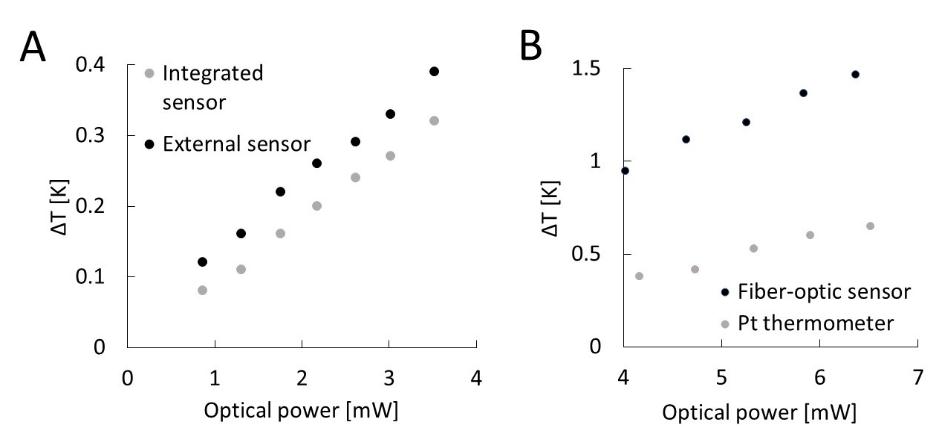


*Figure S3.: Representative curves to compare integrated vs external temperature sensor (a) and external Pt temperature sensor vs fiber-optics based temperature sensor (b).*

Figure S4


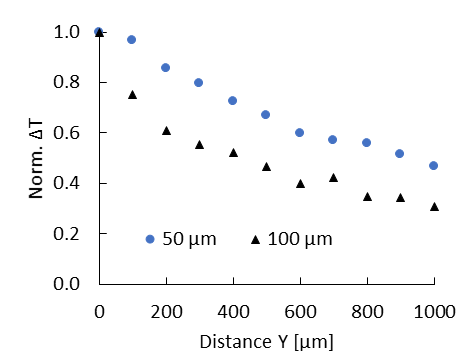


*Figure S4. Effect of fiber core diameter on temperature distribution*

Figure S5


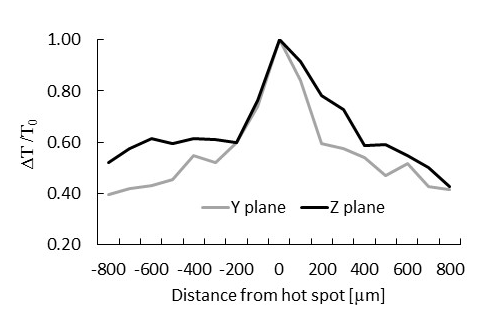


*Figure S5. Normalized spatial distribution of optical heating along two perpendicular axes y and z according to Fig. 2.A.*

Figure S6


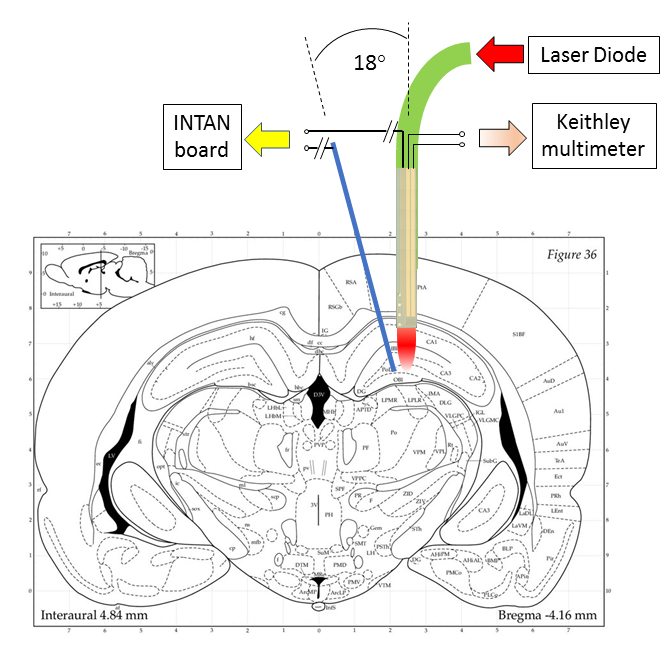


*Figure S6. Experimental arrangement for the in vivo validation of spatially controlled heating in the deep tissue. The blue line represents a silicon probe used as control for the evaluation of electrophysiological response of the heated tissue.*

Figure S7.


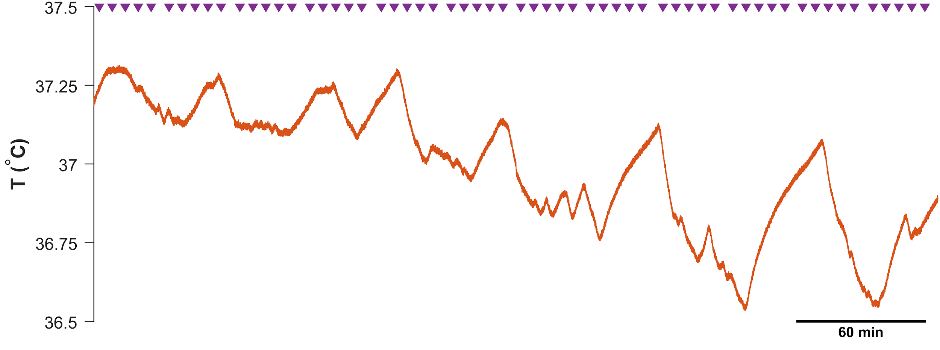


*Figure S7. Body temperature fluctuations monitored during stimulus sequences. We found no correlation between body temperature and brain temperature during the experiments, which confirms that changes in temperature had only local effect inside the brain.*

Figure S8


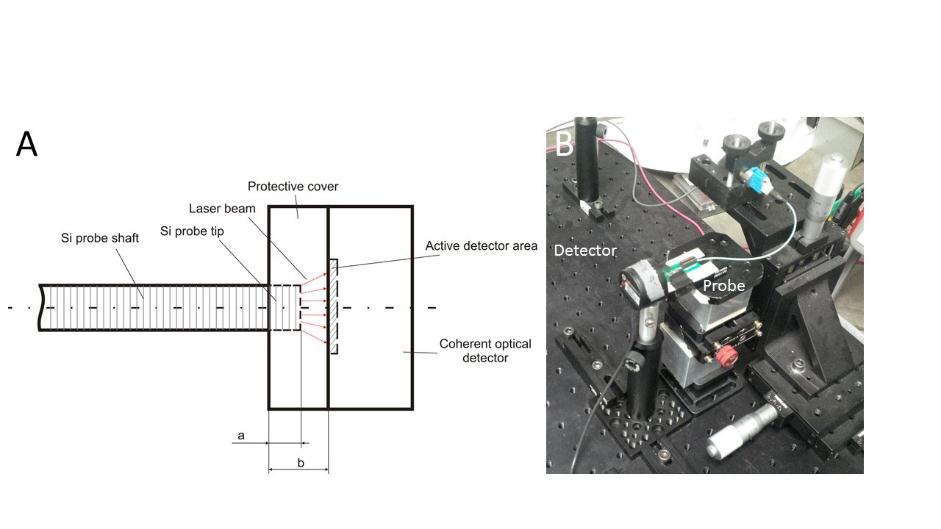


*Figure 8 A: Schematic of the relative position of the optrode’s waveguiding shaft and the IR detector’s active surface. (a=7 mm; b=7.1 mm) B: Photo on optical power measurement.*

Figure S9


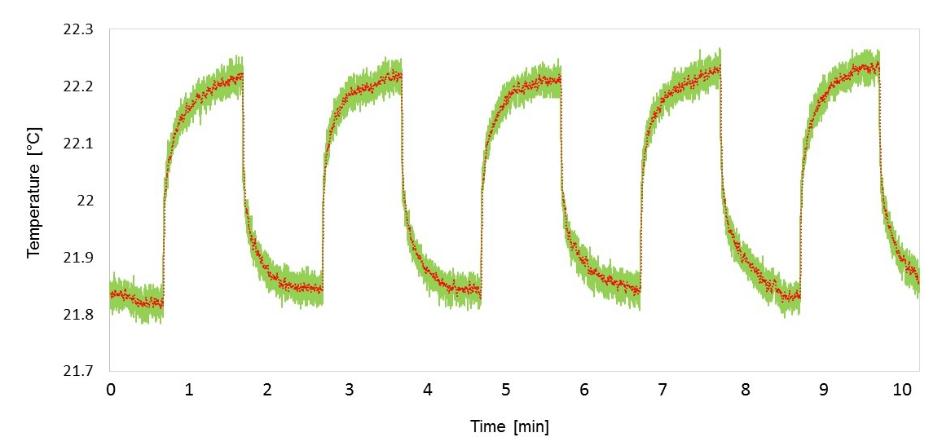


*Figure S9. Time domain representation of recorded data during optically induced heating with cycles of square wave signals (T = 2 min, P = 4.16 mW). The maximum temperature was probed at the plateau of five consecutive cycles to evaluate the average rise in temperature in response at a particular excitation scheme.*

Figure S10


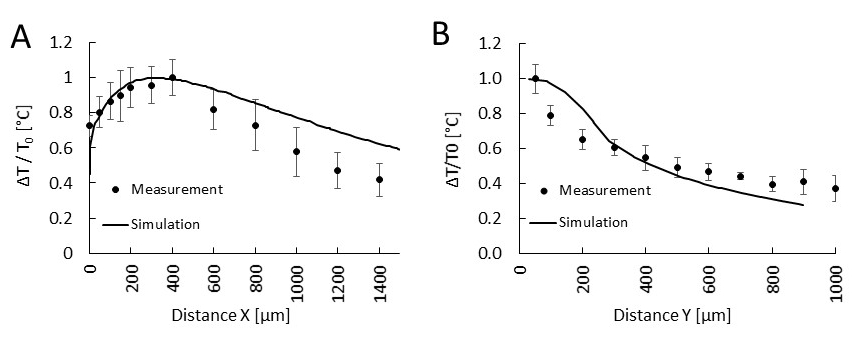


*Figure S10. Measured and simulated temperature in water using 7.88 mW peak value, 120 s periodic time and 60 s pulse length excitation, at stationary state. A: along axes x, B: along axes y (see Figure 2).*

Figure S11


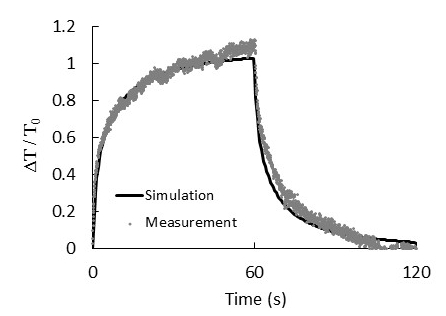


*Figure S11. Measured and simulated temporal change of temperature in water using 2.68 mW peak value, 120 s periodic time and 60 s pulse length excitation.*

Figure S12.

*Figure S12. Difference between simulated and in vivo temperature values induced by various input optical powers. Similarly to the calibration of integrated sensor with an external one, the simulation results have to be also scaled by 24 ± 4 %.*
